# Meta-analysis of the efficacy and safety of rituximab in the treatment of primary Sjögren’s syndrome

**DOI:** 10.3389/fimmu.2025.1561214

**Published:** 2025-06-24

**Authors:** Xiao Zheng, Jiaoyang Di, Xuexiao Chen, Fang Li, Yixuan Liu, Jingjing Cao, Xiaoran Ning, Lin Wei, Guimin Zheng

**Affiliations:** ^1^ Department of Rheumatology and Immunology, Hebei Provincial People’s Hospital, Shijiazhuang, Hebei, China; ^2^ Department of Obstetrical, Hebei Provincial People’s Hospital, Shijiazhuang, Hebei, China; ^3^ Department of Hospital Infection-Control, Hebei Provincial People’s Hospital, Shijiazhuang, Hebei, China

**Keywords:** efficacy, primary Sjögren’s syndrome, rituximab, safety, treatment

## Abstract

**Objective:**

To methodically assess the effectiveness and safety of rituximab in treating primary Sjögren’s syndrome.

**Methods:**

The search included databases such as PubMed, Embase, the Cochrane Library, and Web of Science, covering the period from the beginning until December 2023. A meta-analysis was conducted using Stata 14.0 software.

**Results:**

Five randomized controlled trials (RCTs) with a total of 340 patients were incorporated. The meta-analysis revealed that the group treated with rituximab had significantly lower scores in ESSPRI, pain VAS, serum IgG levels and The blood B cell level was significantly reduced, demonstrating superior results compared to the control group. However, the treatment did not effectively reduce the ESSDAI score, stimulated and unstimulated salivary flow rates and Schirmer’s test results. However, it did lead to a reduction in RF levels and enhanced complement C4 were not statistically different from the values in the control group. The occurrence of adverse events, such as infection, did not show a statistically significant difference between the two groups.

**Conclusion:**

Research has demonstrated that rituximab can decrease the levels of serum IgG and serum B cells in individuals with Sjögren’s syndrome. Additionally, it can significantly enhance the ESSPRI score and pain VAS score in patients. However, it does not have a notable impact on glandular function. The occurrence of adverse effects was like that of the control group. Given the scarcity of research, the findings may be influenced by bias, and a substantial quantity of rigorous investigations is required to validate them.

## 1 Introduction

Sjögren’s syndrome (pSS) is a chronic systemic autoimmune disease characterized by dryness of the mouth and eyes. It can also affect various other systems in the body, including the skin, joints, muscles, lungs, kidneys, nerves, and blood (1–3). SS can either be primary Sjogren syndrome (pSS) or secondary Sjogren syndrome (sSS) when patients have another well defined major connective tissue disease, in particular rheumatoid arthritis and systemic lupus erythematosus (4).This condition significantly impacts the quality of life for patients and can even pose life-threatening risks (5, 6). SS pathogenesis remains unclear, though it is likely a complex process involving genetic factors and environmental triggers that cause an atypical immune response. The strongest link has been found with HLA class II genes like HLA-DQB1 and HLA-DQA1, indicating that presenting antigens to CD4+ T cells, resulting in excessive immune activation, is a key pathogenic mechanism (7, 8). Of note, in individuals with Sjögren’s syndrome, the presence of numerous autoantibodies in the serum indicates an important level of B cell activity. B cells are crucial in the progression and development of pSS (9, 10). Currently, there is no established and efficacious treatment for pSS (11).There is a dearth of effective pharmaceutical treatments supported by evidence-based medicine for many conditions such as dryness, weariness, discomfort, and internal organ damage. Currently, most of the medications utilized are based on empirical treatment approaches or borrowed from the treatment of similar disorders.

The Standard of care (SoC) in systemic autoimmune diseases consists of treatment with corticosteroids and conventional immunosuppressive drugs (12). Among them, rituximab, also known as Rituximab RTX, is a type of monoclonal antibody that is made up of both human and mouse components. It works by triggering an immune response that leads to the destruction of B cells. This is achieved by attaching particularly to a protein called CD20, found on the surface of B cells. It has been extensively utilized in the treatment of rheumatoid arthritis (13) as well as connective tissue illnesses including systemic lupus erythematosus. Since the confirmation by SomerBG (14) and other researchers of the effectiveness of rituximab in treating Sjögren’s syndrome with lymphoma, a significant number of researchers have been actively doing relevant studies on the application of RTX in pSS. However, the current research findings are inconclusive and vary. Some RCTs have reported that there was significant improvement from baseline in fatigue visual analog scale, social functioning in the rituximab group in contrast to the placebo group (15, 16). However, a RCT indicated that rituximab is neither clinically effective nor cost-effective in this patient population (17). Considering the recent progress in using rituximab for pSS, it is both relevant and new to perform a meta-analysis to determine the drug’s efficacy and safety in symptom improvement.

This study aimed to conduct a meta-analysis to summarize and analyze the effectiveness and safety of rituximab in treating primary Sjögren’s syndrome (pSS). The goal was to evaluate the clinical value of rituximab comprehensively and quantitatively in the treatment of pSS.

## 2 Methods

### 2.1 Search strategy

This systematic review and meta-analysis were conducted based on the preferred reporting items for systematic reviews and meta-analyses (PRISMA) guidelines (18). Studies were only selected for inclusion in accordance with the following PICOS criteria. The literature was searched using various databases including PubMed, Embase, Cochrane Library, Web of Science databases. Additionally, hand searched reviews and references were also included. The PubMed search strategy for this condition are: (“sjogren s syndrome”[Title/Abstract] OR “sjogrens syndrome”[Title/Abstract] OR “syndrome sjogren s”[Title/Abstract] OR “sjogren syndrome”[Title/Abstract] OR “sicca syndrome”[Title/Abstract] OR “syndrome sicca”[Title/Abstract]) AND (“Rituximab”[Title/Abstract] OR “cd20 antibody”[Title/Abstract] OR “rituximab cd20 antibody”[Title/Abstract] OR “Mabthera”[Title/Abstract] OR “idec c2b8 antibody”[Title/Abstract] OR “IDEC-C2B8”[Title/Abstract] OR ((“Rituximab”[Supplementary Concept] OR “Rituximab”[All Fields] OR “GP2013”[All Fields] OR “Rituximab”[MeSH Terms]) AND “Antibody”[Title/Abstract]) OR “GP2013”[Title/Abstract] OR “Rituxan”[Title/Abstract]). The searches were done till 10 July 2023.

### 2.2 Inclusion and exclusion criteria

#### 2.2.1 Inclusion criteria

(1) Study type: The included publications were randomized controlled trials published domestically and internationally, written in either Chinese or English. (2) The study aimed to assess the therapeutic efficacy of RTX in pSS, with an observation duration of 24 weeks. (3) Research subjects: The experimental group fulfilled the classification criteria jointly amended by ACR/EULAR in 2010. The control group comprised individuals in good health or had other connective tissue disorders and tended to become easily disoriented. (4) Intervention: The experimental group received either rituximab alone, rituximab in combination with glucocorticoids, or rituximab in combination with glucocorticoids and disease-modifying anti-rheumatic drugs (DMARDs). The control group employed a combination of placebo, glucocorticoids, DMARDs. Some examples of traditional drugs are leflunomide, methotrexate, hydroxychloroquine, and mycophenolate mofetil. (5) Outcome measurements: The primary outcome measures were the effective rate, ESSDAI score, SF-36 score, and VAS score. Secondary outcomes include measurements of unstimulated salivary flow rate, stimulated salivary flow rate, Schirmer’s test, RF levels, complement C4 levels, CD20 levels, and the risk of infection.

#### 2.2.2 Exclusion criteria

(1) Either a control group study was not conducted, or the control group consisted of a single DMARDs medication research. (2) The prescribed amount and frequency of administration is not 1000 mg, once every two weeks. (3) The follow-up period did not encompass the 24-week trial. (4) The complete text and accurate Statistics were not acquired by direct communication with the authors or researching the relevant literature.

### 2.3 Extraction of data

The task is carried out autonomously by two researchers, and in the event of divergent outcomes, it will be addressed through negotiation or consultation with experts. Extracted contents: (1) Research background and design information: includes the research article number, publication time, and research country. It also includes details about the race, mean age, number of participants, male-female ratio, course of disease, diagnostic criteria, interventions used, and observation period. (2) Outcome measures: includes ESSDAI score, SF-36 score, VAS score, unstimulated salivary flow rate, stimulated salivary flow rate, Schirmer test results, RF levels, IgG levels, complement C4 levels, CD20 levels, and any adverse events reported. or by performing calculations to acquire the facts.

### 2.4 Evaluation of research quality

The literature’s quality was assessed using the QUADAS-2, a tool included with Review Manager 5.2 software, which evaluates the quality of diagnostic accuracy studies. The task is carried out autonomously by two researchers, and in the event of disparate outcomes, they will be settled through negotiation or consultation with experts.

### 2.5 Statistical processing

Stata 14.0 software was used for publication bias assessment, heterogeneity testing, data pooling, and funnel and forest plots. A Random-effects model was used to perform a meta-analysis of the included studies. The processed data is continuous data, and the weighted mean difference (WMD) is used when the units are consistent, and the standardized mean difference (SMD) is used to process the units are inconsistent. 0.05 was used as the cut-off point in the effect size. The heterogeneity of the included studies was assessed using household and Cochran’s Q tests. Based on the results, the degree of heterogeneity was classified as non-existent, low, moderate, or high, depending on whether it was less than 25%, between 25% and 50%, between 50% and 75%, or greater than or equal to 75%, respectively. Funnel plots and Egger’s test were used to identify publication bias in the studies included. In the presence of publication bias, the “trim and fill” method was used to assess the combined findings’ robustness.

## 3 Results

### 3.1 Search results

**Figure 1:**
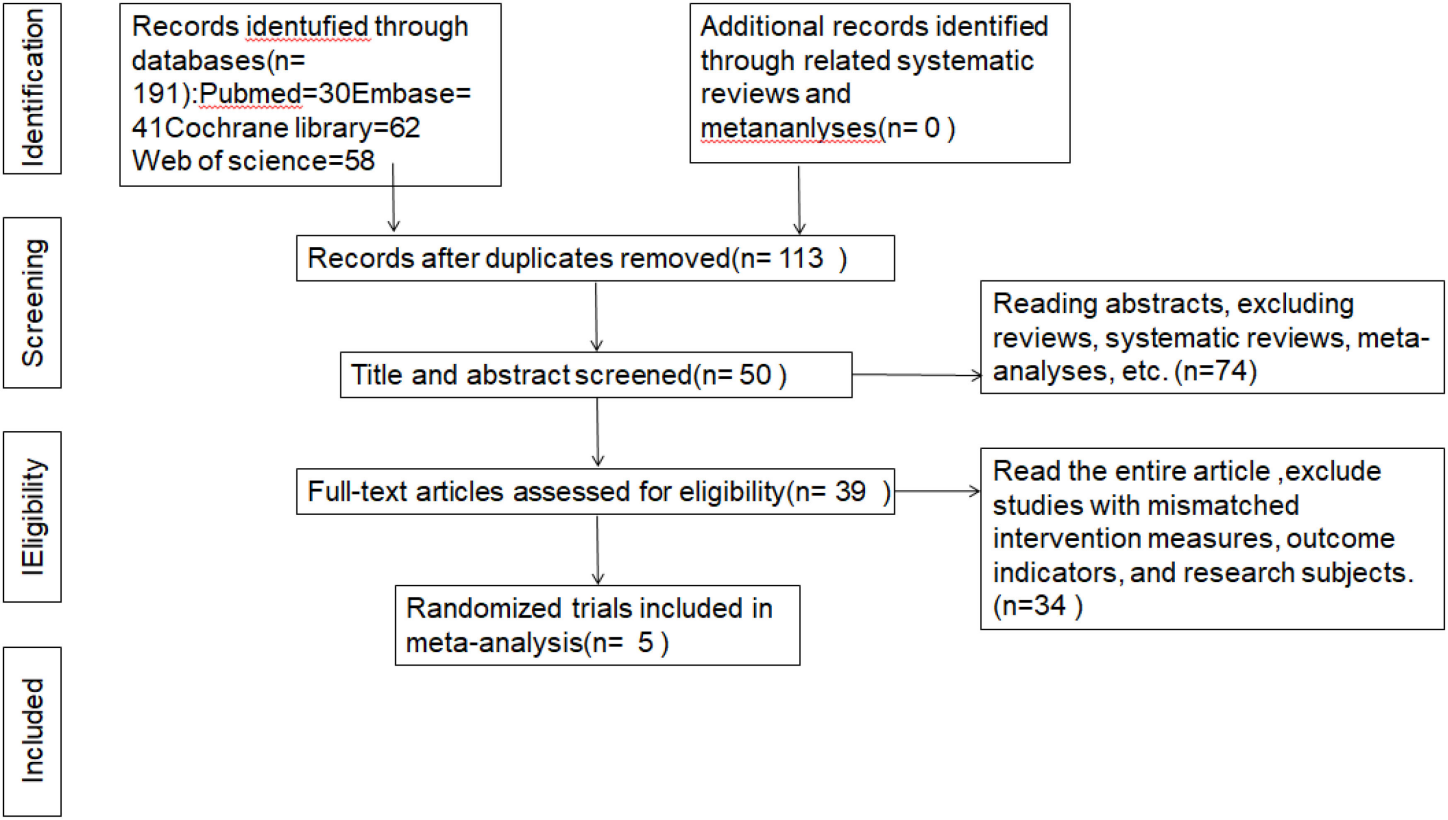
Flow chart of studies selection.

A total of obtained items were retrieved in the initial search. Out of 191 papers, 113 articles remained after removing duplicates based on the title. After further filtering by considering both the article title and abstract, excluding reviews, systematic reviews, and meta-analysis, 39 articles were left. Upon downloading and thoroughly reviewing the complete text, a compilation of 5 publications was identified, omitting literature pertaining to therapies, outcome measures, research participants, and data that was unavailable (15–17, 19, 20). **Figure 1** as PRISMA flowchart to provides details concerning the 5 rationale for rejecting studies.

### 3.2 Literature quality evaluation and basic characteristics

#### 3.2.1 Include the basic characteristics of the literature

This meta-analysis had 338 patients (**Table 1**) with 183 individuals in the experimental group and 155 in the control group. All participants received treatment with standard medications.

**Table 1:** Basic characteristics included in the study.

Studies	Sample size		Age		Course of a disease		Outcome index
	T	C	T	C	T	C	
Mariette et al. (19)	25	13	55.2 ± 15.07	52.7 ± 12.67	6.1 ± 4.32y	8.8 ± 8.61Y	①②⑥⑦⑧⑨ 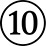 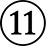 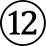
Devauchelle-Pensec et al. (21)	63	57	52.9 ± 13.3	55.6 ± 13.6	4.6 ± 4.8y	5.5 ± 6.5y	①③④⑤⑥⑧⑨ 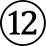
Bowman et al. (17)	67	66	54.3 ± 11.5	54.4 ± 11.6	5.3 ± 4.9y	6.2 ± 5.8y	①②③④⑤⑥ 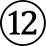
Dass et al. (16)	10	9	51 ± 10.5	51 ± 5.75	7.25 ± 4.25y	8.25 ± 4.25y	③⑤
Meijer et al. (15)	20	10	43.0 ± 11.0	43.0 ± 17.0	67 ± 63m	63 ± 50m	④⑤⑥⑦⑧ 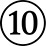 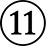

①ESSDAI ②ESSPRI ③fatigue VAS ④oral drynes VAS ⑤SF-36 ⑥unstimulate salivary flow rate ⑦stimulate salivary flow rate ⑧Schirmer test ⑨IgG 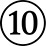
RF 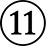
CD19+CD27+Bcells 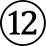
adverse events.

T, test; C, control.

#### 3.2.2 Literature quality evaluation

Review Manager 5.3: The program is included. QUADAS-2, also known as quality evaluation of diagnostic accuracy studies-2, is a tool used to assess the quality of diagnostic accuracy studies. This is a meta statement. An analysis was conducted to evaluate the quality of the studies included. The Cochrane Handbook version 5.1.0 suggests using randomized controlled trials (RCTs).

**Figure 2:**
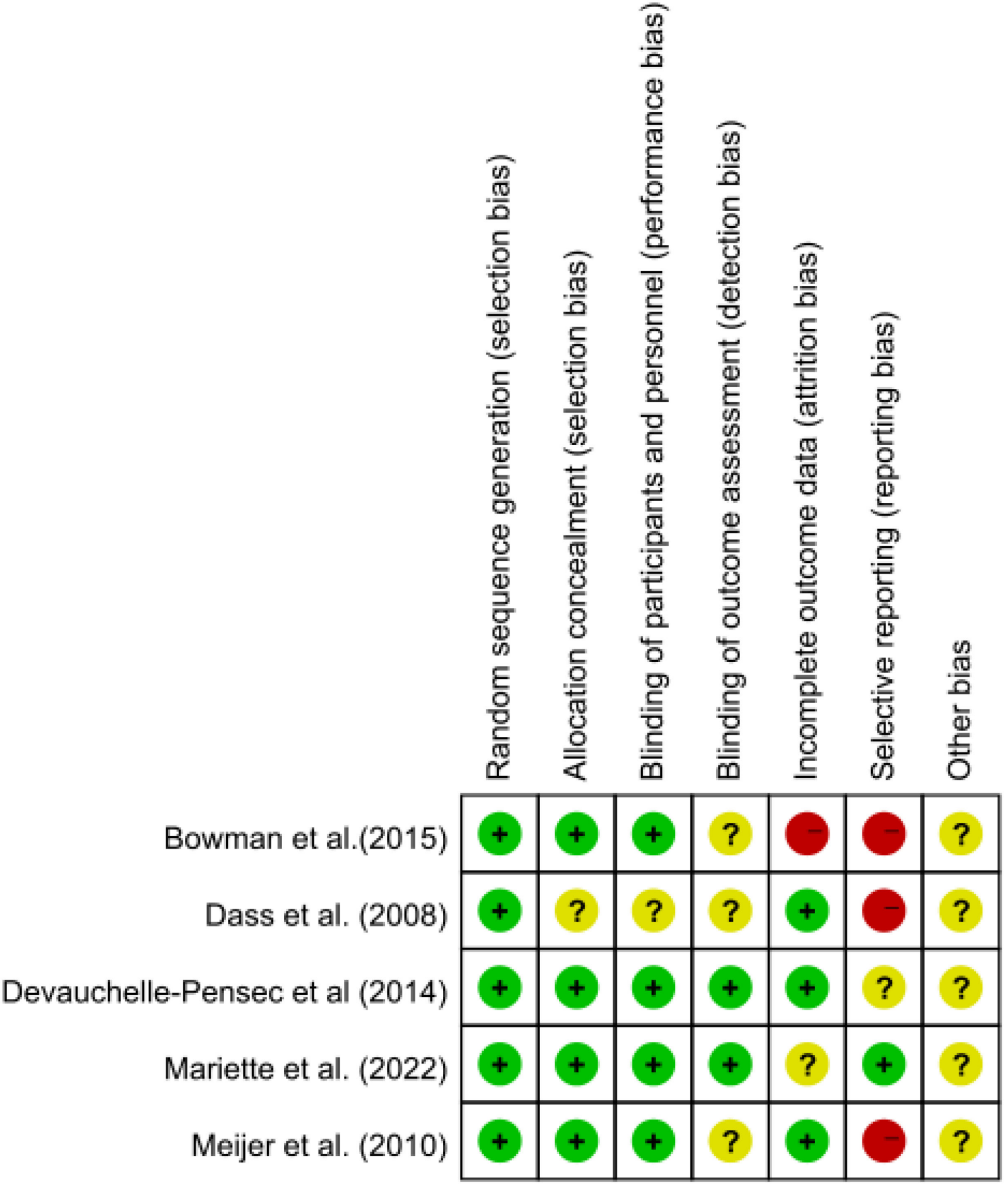
Results of bias risk assessment included in the study.

A bias risk assessment was conducted, and the findings were displayed as a risk of bias map, with green indicating insignificant risk, yellow indicating uncertain danger, and red indicating considerable risk (**Figure 2**).

### 3.3 Efficacy evaluation

#### 3.3.1 ESSDAI score

**Figure 3:**
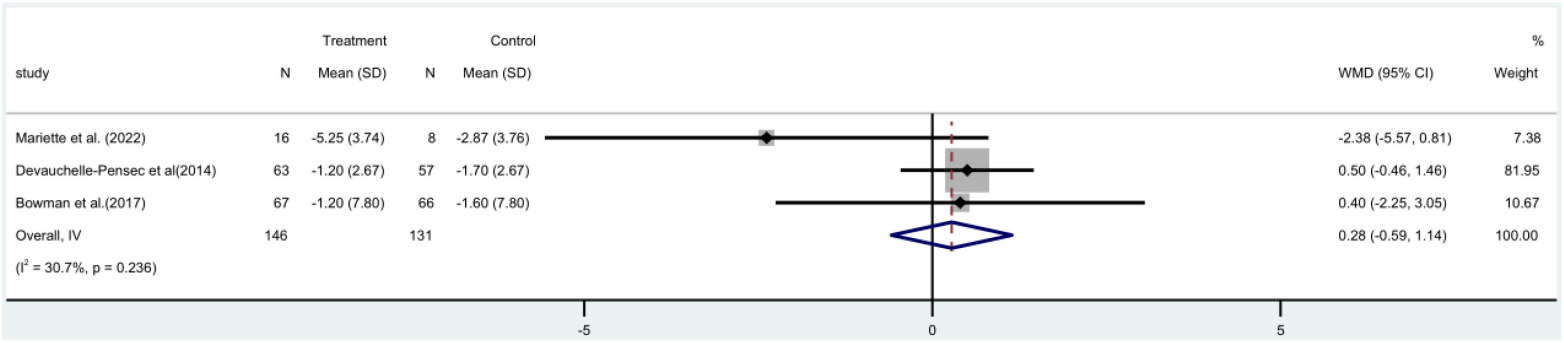
Meta-analysis of the outcome ESSDAI at week 24.

The ESSDAI study incorporated three (17, 19, 21) randomized controlled trials (RCTs), with a combined total of 277 people. A meta-analysis utilizing a fixed-effect model revealed that there was no statistically significant distinction between the rituximab group and the control group in terms of ESSDAI score both before and after treatment [MD=0.28, 95%CI (-0.59, 1.14)]. Additionally, the rituximab group did not demonstrate a significant reduction in patients’ ESSDAI score (**Figure 3**).

#### 3.3.2 ESSPRI score

**Figure 4:**
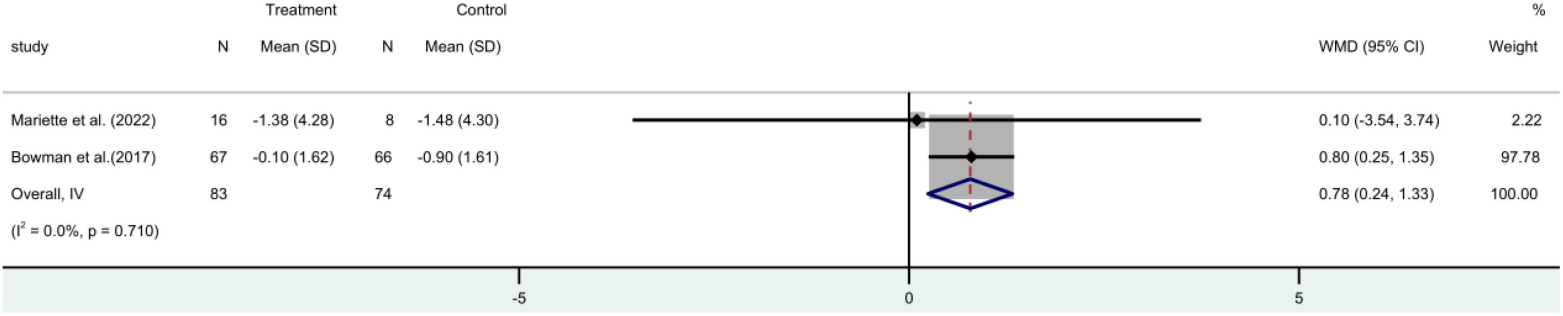
Meta-analysis of the outcome ESSPRI at week 24.

The ESSPRI score incorporated two (17, 19) RCTs. These trials involved 157 participants. A meta-analysis employing a fixed-effect model revealed that the ESSPRI score was significantly reduced in the rituximab group compared to the control group [MD=0.78, 95%CI (0.24 to 1.33)]. This suggests that rituximab treatment can effectively lower the ESSPRI score in patients (**Figure 4**).

#### 3.3.3 VAS score is divided into pain, fatigue, and dry mouth scores

**Figure 5:**
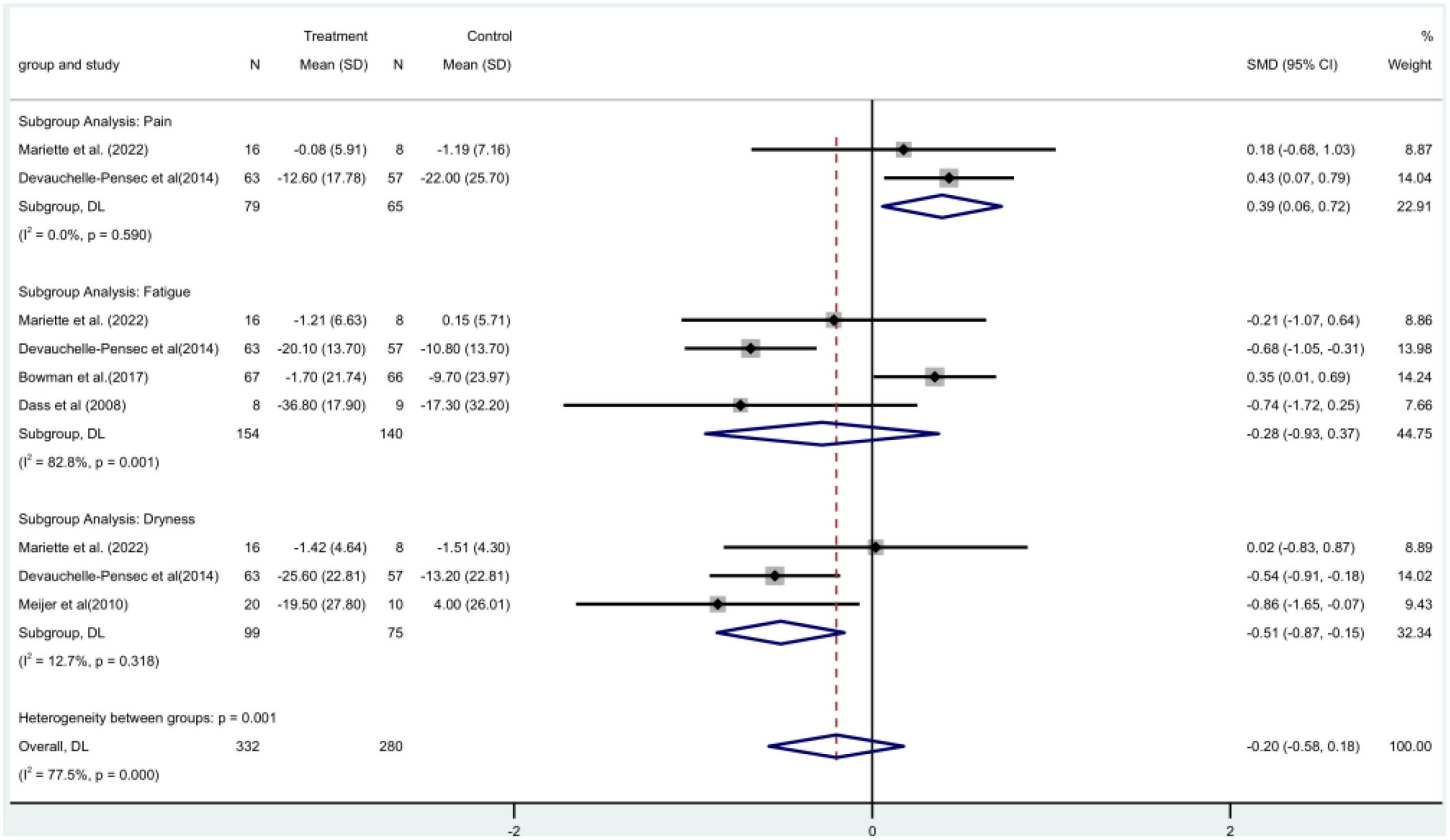
Meta-analysis of the outcome VAS at week 24.

Two (19, 21) RCTs involved a total of 144 patients and assessed pain using the Visual Analog Scale (VAS) score. A meta-analysis using a fixed-effect model revealed that the rituximab group had a larger difference in pain VAS scores before and after treatment. This indicates that rituximab significantly reduced the pain VAS scores of patients, with a statistically significant difference [MD=0.39, 95%CI (0.06, 0.72)]. (**Figure 5**).

VAS fatigue score was included in four RCTs (16, 17, 19, 21) with a total of 294 patients. Meta-analysis using a random-effects model showed that there was no significant difference between the rituximab group and the control group before and after fatigue VAS score [MD = -0.28, 95% CI (-0.93, 0.37)]. The rituximab group did not significantly reduce the VAS fatigue score (**Figure 5**).

Three (15, 19, 21) RCTs with 174 patients in all included oral dryness VAS score. The rituximab group and the control group did not differ significantly before or after the VAS score for dry mouth [MD = -0.51, 95% CI (-0.87, -0.15)] according to a fixed-effect model meta-analysis. The oral dryness VAS score was not much lowered in the rituximab group (**Figure 5**).

### 3.4 SF-36

**Figure 6:**
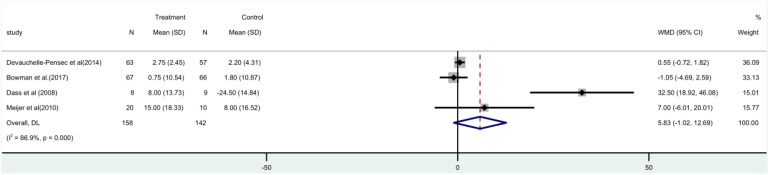
Meta-analysis of the outcome SF-36 at week 24.

The SF-36 score was used in four (15–17, 21) randomized controlled trials, involving 300 patients. A meta-analysis utilizing a random-effects model indicated that there was no substantial disparity observed between the rituximab group and the control group in terms of the SF-36 score, both before and after [MD=5.83, 95%CI (-1.02, 12.69)]. The group treated with rituximab did not show a significant reduction in the SF-36 score (**Figure 6**).

### 3.5 Saliva flow rate:Stimulated saliva flow rate and unstimulated saliva flow rate

**Figure 7:**
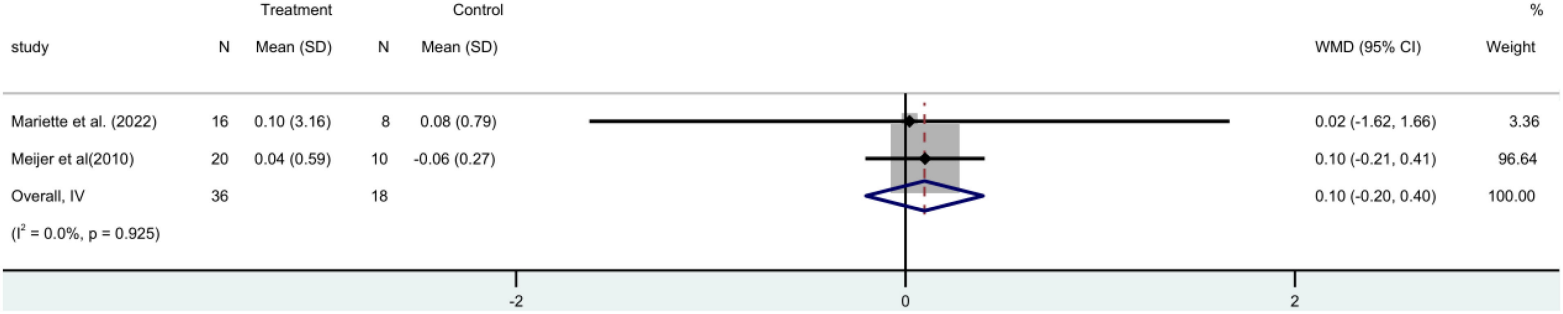
Meta-analysis of the outcome stimulated salivary flow rate at week 24.

Two RCTs (15, 19) with 54 participants in all that stimulated salivary flow rate. A fixed-effect meta-analysis revealed no statistically significant difference between the rituximab and control groups [MD=0.10, 95%CI (-0.20, 0.40)]. Shows that the stimulus flow rate of the patient is not increased by rituximab (**Figure 7**).

**Figure 8:**
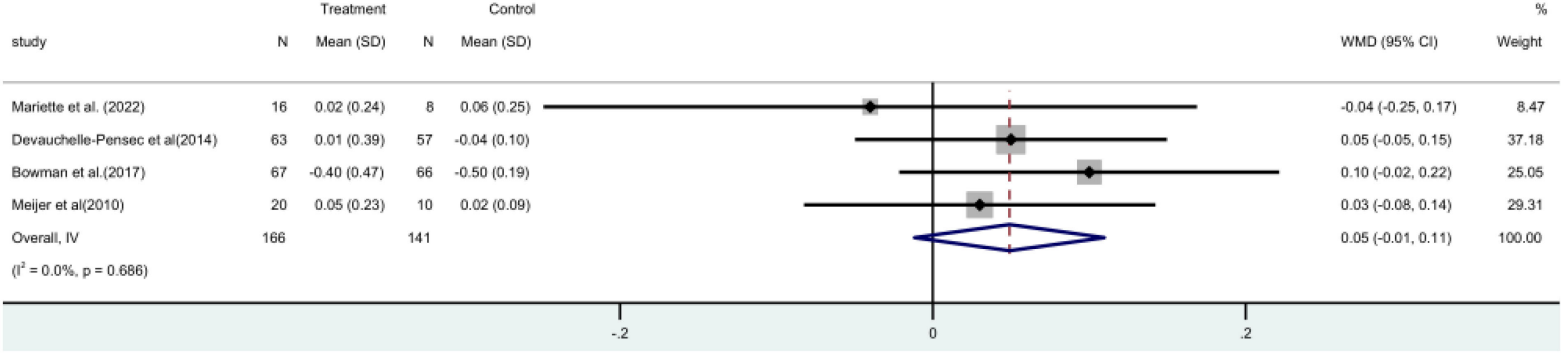
Meta-analysis of the outcome unstimulated salivary flow rate at week 24.

Four (15, 17, 19, 21) randomized controlled trials (RCTs) involving a total of 307 patients, measured the unstimulated salivary flow rate. A meta-analysis using a fixed-effect model revealed that there was no statistically significant distinction observed between the two groups [MD = 0.05, 95% CI (-0.01, 0.11)]. It is evident that rituximab does not have a positive impact on salivary flow rate (**Figure 8**).

### 3.6 The Schirmer trial

**Figure 9:**
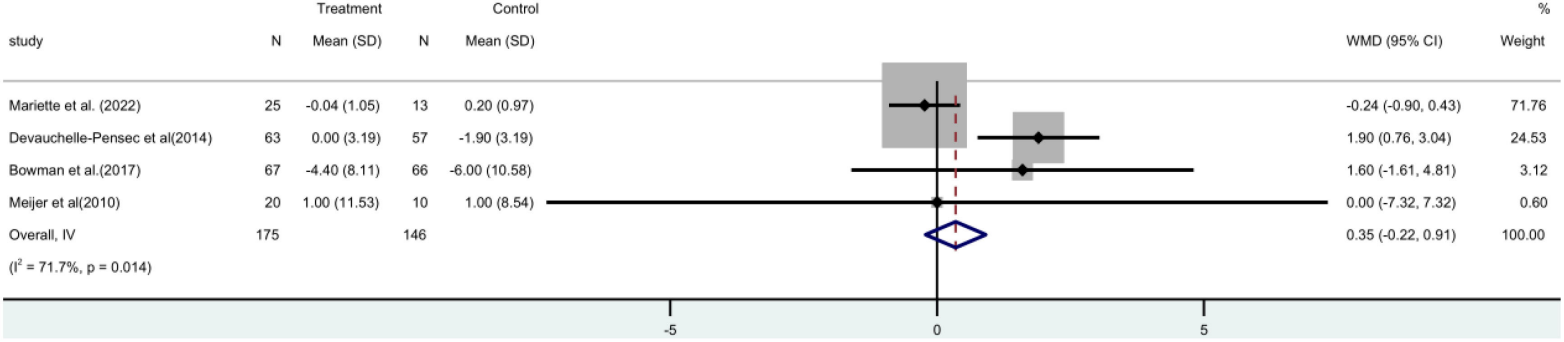
Meta-analysis of the outcome Schirmer trial at week 24.

The Schirmer trial consisted of four (15, 17, 19, 21) randomized controlled trials (RCTs), involving a total of 312 patients. A meta-analysis utilizing a random-effects model revealed no statistically significant distinction between the two groups [MD=0.35, 95% CI (-0.22, 0.91)]. It has been found that rituximab does not have a positive impact on tear gland function in patients (**Figure 9**).

### 3.7 Immunoglobulin IgG

**Figure 10:**
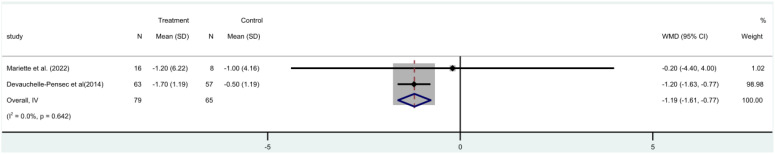
Meta-analysis of the outcome IgG at week 24.

Two (19, 21) randomized controlled trials (RCTs) were conducted, involving 144 patients, IgG was included as a factor in these trials. A meta-analysis utilizing a fixed-effect model revealed a significant decrease in IgG levels within the rituximab group when compared to the control group [MD = -1.19, 95% CI (-1.61 to -0.77)]. There was no discernible distinction in IgG levels between the two (**Figure 10**).

### 3.8 RF

**Figure 11:**
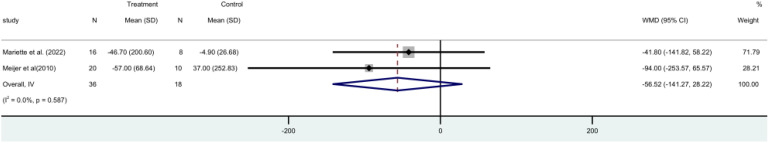
Meta-analysis of the outcome RF at week 24.

RF ratings were used in two RCTs (15, 19) included 54 participants in all. A fixed-effect model meta-analysis revealed no statistically significant difference [MD = -56.52, 95% CI (-141.27, 28.22)] between the two groups. The group using rituximab did not lower RF. levels (**Figure 11**).

### 3.9 C4

**Figure 12:**
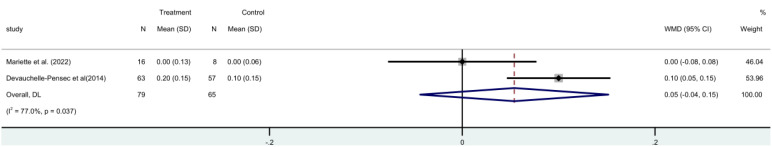
Meta-analysis of the outcome C4 at week 24.

The C4 score comprised two RCTs (19, 21) with 144 patients in all. The two groups did not differ statistically significantly [MD=0.05, 95%CI (-0.04, 0.15)] according to a random-effects meta-analysis. The findings showed that the rituximab group was unable to raise C4 levels (**Figure 12**).

### 3.10 B cells

**Figure 13:**
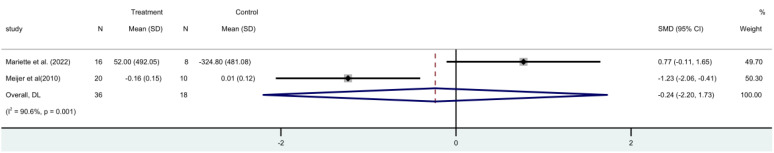
Meta-analysis of the outcome B cell at week 24.

B cells were included in two (15, 19) randomized controlled trials (RCTs), respectively, involving a total of 54 patients. A meta-analysis utilizing a random-effects model revealed that there was no statistically significant disparity between the two groups [MD)= -1.29, 95%CI (-1.91, -0.67)]. The findings demonstrated that the group treated with rituximab exhibited a substantial decrease in the concentration of B cells (**Figure 13**).

### 3.11 Infection risk assessment

In order to evaluate the likelihood of infection, three RCTs (15, 19, 21) were analyzed, involving a total of 402 patients. The meta-analysis, employing a fixed-effect model, demonstrated that there was no statistically significant distinction between the two groups [MD = 1.00, 95% CI (0.61, 1.63)]. There was no notable disparity in the risk of infection between the two groups.

## 4 Discussion

Patients with primary Sjogren’s syndrome (pSS) have multiple autoantibodies in their bodies, such as SS-A (Ro), SS-B (La), and rheumatoid factor (RF), as well as abnormal lymphoid tissue development in exocrine glands. This evidence suggests that B cells have a significant impact on the pathogenesis of pSS (22). Rituximab is a chimeric monoclonal antibody that targets the CD20 antigen on B cells, triggering an immunological response and causing B cell destruction. In theory, rituximab has shown potential as a treatment for pSS. Clinical practice has demonstrated positive outcomes for some patients with pSS, particularly those with severe cases or lymphoma. The European League Against Rheumatism (EULAR) 2019 has developed recommendations for the treatment of Sjögren’s syndrome, both topically and systemically. It suggests that B-cell targeting could be an option for patients with severe refractory systemic disease associated with primary Sjögren’s syndrome (23). This meta-analysis confirmed that patients with pSS underwent a 24-week treatment, there was a considerable drop in the number of B cells. Nevertheless, the specific time frame for administering rituximab as a treatment for pSS has not been established, and the duration of observation differs across different studies. The trials published observational data at 12 weeks, 24 weeks, and 36 weeks, indicating that B lymphocytes were significantly reduced at 12 weeks and did not return to their initial levels by 36 weeks. This study will provide valuable references for tailoring the dose interval of rituximab in the treatment of pSS.

The levels of RF and IgG in individuals with pSS were predominantly elevated, with IgG being identified as a marker for assessing the activity of Sjögren’s syndrome, which is directly associated with cutaneous vasculitis. A meta-analysis has been verified, Rituximab has a notable effect in decreasing blood IgG levels and alleviating the symptoms of vasculitis in patients.

Clinically, a sizable portion of pSS patients just experience dry mouth and eyes, which show up as frequent thirst, trouble swallowing dry food, and even impairment of regular speech. Effective therapeutic medications can enhance patients’ quality of life. Since its discovery in 1996 (14), rituximab has shown promising results in improving glandular function in individuals with Sjögren’s syndrome (SS) associated with lymphoma. However, subsequent investigations on the use of rituximab in primary Sjögren’s syndrome (pSS) have yielded poor outcomes (17). This contradictory outcome raises questions about its application in clinical environments. Thus, this meta-analysis examined five studies that analyzed the Schirmer trial to assess lacrimal gland function. Among these studies, two (10, 11) conducted demonstrated effectiveness in improving lacrimal gland function. The other studies concluded that rituximab was not effective in improving lacrimal gland function. Based on a meta-analysis, it is contended that Rituximab does not enhance lacrimal gland function. Similarly, medicine did not effectively improve the salivary gland function as assessed by the irritating salivary flow rate and the unstimulated salivary flow rate.

The ESSDAI index has been extensively utilized in clinical and scientific investigations as a metric for assessing the disease activity of primary Sjögren’s syndrome (pSS). The research using the fixed-effect model in this article found that rituximab did not have a significant improvement on the ESSDAI score, is same with Souza (24).In contrast, RTX is widely utilized in clinical practice to treat refractory pSS with multiple organ involvement (25). In contrast to clinical conclusions, the reasons for consideration could be connected to the duration of the disease, systemic involvement, and level of activity of the individuals included in the studies. Furthermore, meta-analyses indicate that VAS values for pain in patients with pSS can be significantly improved, but not for weariness or dry mouth. In addition, previous study has reported that rituximab is typically administered via intravenous infusion, which poses a risk of infusion-related reactions. However, a subcutaneous route has been proposed as a more convenient, cost-effective alternative that could improve ease of administration for patients (26). Thus, the method of administration can lead to different clinical outcomes, affecting the effectiveness and safety of treatments.

Of course, this study has some limitations. First, this study is only use 5 RCTs with th total 340 numbers of patient, and each studies has different time to follow up so can affecting the comparability. Second, a limited sample size and certain publication biases could have influenced the outcomes of this study. Third, several factors (e.g., genetics, age, and sex) that influence the outcome of rituximab on treatment for pSS. Further experimental verification is required to confirm the effectiveness and safety of rituximab in treating pSS. In addition, future trials should examine the role of rituximab in patients with pSS in an RCT with a larger sample size, well-controlled confounding factors, sufficient follow-up time, and more accurate assessment of rituximab exposure levels.

## 5 Conclusion

In summary, this study has demonstrated that rituximab can decrease the levels of serum IgG and serum B cells in individuals with Sjögren’s syndrome. Additionally, it can significantly enhance the ESSPRI score and pain VAS score in patients. However, it does not have a notable impact on glandular function. The occurrence of adverse effects was like that of the control group. Taken together, rituximab is clinically effective treatment in this patient population.

## Data Availability

The original contributions presented in the study are included in the article/supplementary material. Further inquiries can be directed to the corresponding author.

## References

[1] Del Papa N Vitali C . Management of primary Sjögren’s syndrome: recent developments and new classification criteria. Ther Adv Musculoskelet Dis. (2018) 10:39–54. doi: 10.1177/1759720x17746319 29387177 PMC5784475

[2] Mariette X Criswell LA . Primary sjögren’s syndrome. N Engl J Med. (2018) 378:931–9. doi: 10.1056/NEJMcp1702514 29514034

[3] Omdal R Mellgren SI Norheim KB . Pain and fatigue in primary Sjögren’s syndrome. Rheumatol (Oxford). (2021) 60:3099–106. doi: 10.1093/rheumatology/kez027 30815693

[4] Ng ALK Choy BNK Chan TCY Wong IYH Lai JSM Mok MY . Comparison of tear osmolarity in rheumatoid arthritis patients with and without secondary sjogren syndrome. Cornea. (2017) 36:805–9. doi: 10.1097/ico.0000000000001227 28486313

[5] Parisis D Chivasso C Perret J Soyfoo MS Delporte C . Current state of knowledge on primary sjögren’s syndrome, an autoimmune exocrinopathy. J Clin Med. (2020) 9. doi: 10.3390/jcm9072299 PMC7408693 32698400

[6] Tian Y Yang H Liu N Li Y Chen J . Advances in pathogenesis of sjögren’s syndrome. J Immunol Res. (2021) 2021:5928232. doi: 10.1155/2021/5928232 34660815 PMC8516582

[7] Thorlacius GE Björk A Wahren-Herlenius M . Genetics and epigenetics of primary Sjögren syndrome: implications for future therapies. Nat Rev Rheumatol. (2023) 19:288–306. doi: 10.1038/s41584-023-00932-6 36914790 PMC10010657

[8] Maleki-Fischbach M Kastsianok L Koslow M Chan ED . Manifestations and management of Sjögren’s disease. Arthritis Res Ther. (2024) 26:43. doi: 10.1186/s13075-024-03262-4 38331820 PMC10851604

[9] Le Pottier L Devauchelle V Fautrel A Daridon C Saraux A Youinou P . Ectopic germinal centers are rare in Sjogren’s syndrome salivary glands and do not exclude autoreactive B cells. J Immunol. (2009) 182:3540–7. doi: 10.4049/jimmunol.0803588 19265132

[10] Jonsson MV Szodoray P Jellestad S Jonsson R Skarstein K . Association between circulating levels of the novel TNF family members APRIL and BAFF and lymphoid organization in primary Sjögren’s syndrome. J Clin Immunol. (2005) 25:189–201. doi: 10.1007/s10875-005-4091-5 15981083

[11] Leverenz DL St Clair EW . Recent advances in the search for a targeted immunomodulatory therapy for primary Sjögren’s syndrome. F1000Res. (2019) 8. doi: 10.12688/f1000research.19842.1 PMC6719673 31508200

[12] Marinho A Delgado Alves J Fortuna J Faria R Almeida I Alves G . Biological therapy in systemic lupus erythematosus, antiphospholipid syndrome, and Sjögren’s syndrome: evidence- and practice-based guidance. Front Immunol. (2023) 14:1117699. doi: 10.3389/fimmu.2023.1117699 37138867 PMC10150407

[13] Mease PJ Revicki DA Szechinski J Greenwald M Kivitz A Barile-Fabris L . Improved health-related quality of life for patients with active rheumatoid arthritis receiving rituximab: Results of the Dose-Ranging Assessment: International Clinical Evaluation of Rituximab in Rheumatoid Arthritis (DANCER) Trial. J Rheumatol. (2008) 35:20–30. doi: 10.1080/10582450802479693 18050385

[14] Somer BG Tsai DE Downs L Weinstein B Schuster SJ . Improvement in Sjögren’s syndrome following therapy with rituximab for marginal zone lymphoma. Arthritis Rheum. (2003) 49:394–8. doi: 10.1002/art.11109 12794796

[15] Meijer JM Meiners PM Vissink A Spijkervet FK Abdulahad W Kamminga N . Effectiveness of rituximab treatment in primary Sjögren’s syndrome: a randomized, double-blind, placebo-controlled trial. Arthritis Rheum. (2010) 62:960–8. doi: 10.1002/art.27314 20131246

[16] Dass S Bowman SJ Vital EM Ikeda K Pease CT Hamburger J . Reduction of fatigue in Sjögren syndrome with rituximab: results of a randomised, double-blind, placebo-controlled pilot study. Ann Rheum Dis. (2008) 67:1541–4. doi: 10.1136/ard.2007.083865 18276741

[17] Bowman SJ Everett CC O’Dwyer JL Emery P Pitzalis C Ng WF . Randomized controlled trial of rituximab and cost-effectiveness analysis in treating fatigue and oral dryness in primary sjögren’s syndrome. Arthritis Rheumatol. (2017) 69:1440–50. doi: 10.1002/art.40093 28296257

[18] Moher D Liberati A Tetzlaff J Altman DG . Preferred reporting items for systematic reviews and meta-analyses: the PRISMA statement. BMJ. (2009) 339:b2535. doi: 10.1136/bmj.b2535 19622551 PMC2714657

[19] Mariette X Barone F Baldini C Bootsma H Clark KL De Vita S . A randomized, phase II study of sequential belimumab and rituximab in primary Sjögren’s syndrome. JCI Insight. (2022) 7. doi: 10.1172/jci.insight.163030 PMC9746921 36477362

[20] Saraux A Nowak E Devauchelle-Pensec V . Treatment of primary Sjögren syndrome with rituximab. In response. Ann Intern Med. (2014) 161:377–8. doi: 10.7326/l14-5017-4 25178575

[21] Devauchelle-Pensec V Mariette X Jousse-Joulin S Berthelot JM Perdriger A Puéchal X . Treatment of primary Sjögren syndrome with rituximab: a randomized trial. Ann Intern Med. (2014) 160:233–42. doi: 10.7326/m13-1085 24727841

[22] Nocturne G Mariette X . B cells in the pathogenesis of primary Sjögren syndrome. Nat Rev Rheumatol. (2018) 14:133–45. doi: 10.1038/nrrheum.2018.1 29416129

[23] Ramos-Casals M Brito-Zerón P Bombardieri S Bootsma H De Vita S Dörner T . EULAR recommendations for the management of Sjögren’s syndrome with topical and systemic therapies. Ann Rheum Dis. (2020) 79:3–18. doi: 10.1136/annrheumdis-2019-216114 31672775

[24] Souza FB Porfírio GJ Andriolo BN Albuquerque JV Trevisani VF . Rituximab effectiveness and safety for treating primary sjögren’s syndrome (pSS): systematic review and meta-analysis. PloS One. (2016) 11:e0150749. doi: 10.1371/journal.pone.0150749 26998607 PMC4801187

[25] Torgashina AV Vasilyev VI . The efficacy of rituximab in the therapy of neuromyelitis optica in a patient with Sjogren’s syndrome: case-report and literature review. Ter Arkh. (2018) 90:76–80. doi: 10.26442/terarkh201890576-80 30701893

[26] Shrivastava P Mariam S Abid L Buch SA Ahmad SA Mansoori S . Rituximab in childhood and juvenile pemphigus vulgaris: A systematic review. Cureus. (2024) 16:e58288. doi: 10.7759/cureus.58288 38752055 PMC11094568

